# *Akkermansia muciniphila*: is it the Holy Grail for ameliorating metabolic diseases?

**DOI:** 10.1080/19490976.2021.1984104

**Published:** 2021-10-21

**Authors:** Juan Yan, Lili Sheng, Houkai Li

**Affiliations:** Institute of Interdisciplinary Integrative Medicine Research, Shanghai University of Traditional Chinese Medicine, Shanghai, China

**Keywords:** *Akkermansia muciniphila*, metabolic diseases, pasteurized *A. muciniphila*, Amuc_1100, AmEVs, P9

## Abstract

The increasing prevalence of metabolic diseases has become a severe public health problem. Gut microbiota play important roles in maintaining human health by modulating the host’s metabolism. Recent evidences demonstrate that *Akkermansia muciniphila* is effective in improving metabolic disorders and is thus considered as a promising “next-generation beneficial microbe”. In addition to the live *A. muciniphila*, similar or even stronger beneficial effects have been observed in pasteurized *A. muciniphila* and its components, including the outer membrane protein Amuc_1100, *A. muciniphila*-derived extracellular vesicles (AmEVs), and secreted protein P9. Hence, this paper presents a systemic review of recent progress in the effects and mechanisms of *A. muciniphila* and its components in the treatment of metabolic diseases, including obesity, type 2 diabetes mellitus, cardiovascular disease, and nonalcoholic fatty liver disease, as well as perspectives on its future study.

## 1. Introduction

The prevalence of metabolic diseases such as obesity, type 2 diabetes mellitus (T2DM), cardiovascular diseases (CVD), and nonalcoholic fatty liver disease (NAFLD) has becoming a severe public health problem:^1^^, 2^ Gut microbiota can regulate host metabolism by influencing immune maturation and homeostasis, protecting against pathogen overgrowth, regulating intestinal endocrine functions and neurologic signaling, modulating energy metabolism, and producing functional metabolites.^3,4^ The compositional and metabolic changes of intestinal microbiota (dysbiosis) is closely involved in the occurrence of metabolic diseases.^5,6^ Thus, considerable attention has been paid to gut microbiota-targeted therapies on metabolic diseases with diverse approaches, including probiotics, prebiotics, fecal microbiota transplantation, and antibiotics.^7–12^

*Akkermansia muciniphila*, a commensal bacterium, is an oval-shaped, non-motile bacterium with no endospore formation, and a microaerophilic microbe that was first isolated from human feces in 2004.^13–15^ It colonizes in the intestinal tract early in life, and accounts for approximately 1–3% of the total intestinal microbiota in healthy adults.^16^ *A. muciniphila* resides in the intestinal mucus layer, utilizing the mucin as the sole source of carbon, nitrogen, and energy.^13^ In recent years, *A. muciniphila* has attracted much attention for its comprehensive roles in maintaining host wellbeing,^17^ which is regarded as a promising “next-generation beneficial microbe” for metabolic disease prevention or therapy owing to its various properties, including producing short-chain fatty acids (SCFAs),^13,15,18^ improving intestinal integrity,^19,20^ and reducing endotoxemia through inhibiting the translocation of lipopolysaccharide (LPS) from the intestine to circulation.^19–21^ However, the exact mechanisms underlying the benefits of *A. muciniphila* on metabolic diseases are complicated because similar effects have been observed on either live *A. muciniphila* or pasteurized *A. muciniphila*, their outer membrane proteins or secreted proteins, as well as extracellular vesicles.^19–42^ In these papers, we systemically reviewed the updated progress of *A. muciniphila* with respect to its role in metabolic diseases, and discussion on its future research direction as well.

## 2. Role of *A. muciniphila* in metabolic diseases

### 2.1. A. muciniphila *and obesity*

In recent years, with the changes in people’s dietary habits and the increased availability of high calorie diet, overweightness and obesity have become one of the most serious health problems in the world. Manipulation of gut microbiota is a promising strategy for obesity prevention or therapy.^43–46^ The relative abundance of *A. muciniphila* is significantly reduced in high-fat diet (HFD)-fed obese mice or rats compared with their lean littermates, and negatively correlated with body fat mass and glucose intolerance (Table 1).^19,47–52^ Nevertheless, inconsistent results have also been reported. For example, the copy number of *A. muciniphila* had an increased trend in high-fat and high-sucrose diet (HFHS)-fed mice without reaching a statistical significance when analyzed by qPCR.^53^ In addition, by 16s rRNA sequencing, Arias *et al*. found significantly increased relative abundance of *Akkermansia* genus in HFD-fed female C3HeB/FeJ mice.^54^ Interestingly, the dietary impacts on *Akkermansia* levels were found to be genetically dependent in mice: *Akkermansia* was decreased in 129S1/SvImJ mice, but was increased in A/J, NOD/LtJ, C57BL/6 J, and NZO/HILtJ mice after short-term HFHS intake.^55^ Thus, the divergent genetically susceptible to intestinal microenvironment might account for the different response of *A. muciniphila* abundance to diet in the host. In clinical studies, a decreased abundance of *A. muciniphila* occurred in adults and children with obesity of both sexes.^56–68^ Furthermore, studies indicated that the reduced *A. muciniphila* in patients with obesity was independent of other metabolic diseases, including diabetes and NAFLD.^65,66,68,69^ In contrast, an increase in *A. muciniphila* abundance was observed in children with obesity compared to children with normal-weight.^70^ Despite some inconsistent findings, the majority of studies in both animal and clinical studies support the negative correlation between *A. muciniphila* and obesity.

**Table 1.** The differential changes of *A. muciniphila* in metabolic diseases

Ref.	Disease condition	Sample type	Sample detection	Study group	Beneficial changes achieved
Mice					
Everard *et al* (2013)^19^	Obesity	Cecal sample	qPCR	1. ob/ob mice (*n* = 5) 2. High fat-fed obese mice (*n* = 10)3. Lean littermates (*n* = 5)	The abundance of *A. muciniphila* was 3,300-fold lower in leptin-deficient obese mice than in their lean littermates. It was also observed that a 100-fold decrease of this bacterium in high fat-fed mice.
Everard *et al* (2014)^147^	HFD induced obesity	Cecal sample	Metagenomic sequencing	1. Control diet (*n* = 9)2. HFD (*n* = 7)	HFD treatment profoundly affected the abundance of *Verrucomicrobia*. Meanwhile, *Akkermansia* was not detectable under HFD treatment but was detected in control diet-fed mice.
Schneeberger *et al* (2015)^148^	Diet-induced obesity	Cecal sample, at 3, 6, 12 and 16 weeks	qPCR	1. Chow diet (*n* = 6 mice/diet/time point) 2. High-fat diet (*n* = 6 mice/diet/time point)	The abundance of *A. muciniphila* progressively decline with prolonged dietary treatment in chow diet fed mice, and that this effect is exacerbated upon HFD.
Hussain *et al* (2016)^47^	HFD induced obesity	Feces	qPCR	1. Normal chow diet (NCD) (*n* = 7)2. High fat diet (HFD) (*n* = 7)	The relative abundance of *A. muciniphila* was lower in HFD group than that in NCD group.
Li *et al* (2016)^20^	Atherosclerosis	Feces, at a selected time point	qPCR	1. Normal chow diet (NCD) (*n* = 8–10)2. Daily oral gavage with live *A. muciniphila* (WD+AKK) (*n* = 8–10)3. Heat-killed *A. muciniphila* (WD+hk-AKK) (*n* = 8–10)	The fecal abundance of *A. muciniphila* was significantly reduced by western diet.
Mehrpouya-Bahrami *et al* (2017)^149^	Diet-induced obesity	Feces	qPCR	1. Low fat diet (LFD) (*n* = 10)2. High fat diet (HFD) (*n* = 10)	The abundance of *A. muciniphila* was lower in HFD fed mice than that in LFD fed mice.
Lee *et al* (2018)^48^	Diet-induced obesity	Cecal sample	16S rRNA sequencing, qPCR	1. Normal diet (*n* = 5)2. HFD (*n* = 5)	Compared with normal diet group mice, the relative abundance of *A. muciniphila* was lower in HFD group.
Natividad *et al* (2018)^49^	HFD induced metabolic dysfunctions	Feces	16S rRNA sequencing	1. Control diet (CD) (*n* = 5–6)2. High fat diet enriched with milk-fat (*n* = 5–6)	Compared to CD, HFD-fed mice had lower abundance of bacteria belonging to the *Akkermansia muciniphila* species.
Villamil *et al* (2018)^150^	HFD	Feces, T1 = at weaning, T2 = after eight weeks of dietary intervention	16S rRNA sequencing	1. Control diet (*n* = 12)2. HFD (*n* = 12)	The HFD diet decreased the level of *A. muciniphila.*
Hänninen *et al* (2018)^28^	T1DM	Feces or cecal and colon content	16S rRNA sequencing	1. NOD/MrkTac (*n* = 6) 2. NOD/Jax (*n* = 6)	NOD/MrkTac mice develop diabetes less often and later than NOD/Jax mice because their microbiota is more diverse and more favorably balanced. The level of *A. muciniphila* was lower in NOD/Jax mice than that in NOD/MrkTac. *Akkermansia* may defer diabetes development in NOD/MrkTac mice.
Arias *et al* (2019)^54^	HFD induced obesity	Cecal sample	16S rRNA sequencing	1. Normal chow diet (NCD) (*n* = 20)2. HFD (*n* = 20)	The relative abundance of *A. muciniphila* was higher in HFD group than that in NCD group.
Anhê *et al* (2019)^53^	High fat/high sucrose (HFHS) induced obesity	Feces	qPCR	1. Chow-fed (Chow, *n* = 9) 2. HFHS (*n* = 11)	When compared with the chow-fed group, the copy number of *A. muciniphila* had an increased trend in HFHS group without reaching a statistical significance.
Wang *et al* (2019)^50^	HFD induced obesity	Feces	16S rRNA sequencing, qPCR	1 Normal control (NC) (*n* = 6)2 HFD (*n* = 6)	The abundance of *A. muciniphila* was significantly lower in the HFD group than in NC group.
Yang *et al* (2019)^51^	HFD-induced cognitive deficits	Feces	16S rRNA sequencing, qPCR	1. Chow diet (*n* = 8)2. HFD (*n* = 8)	The relative abundance of *A. muciniphila* was lower in HFD group than that in chow diet group.
Fujisaka *et al* (2020)^151^	HFD induced obesity	Feces	qPCR	1. Normal chow diet (*n* = 4)2. HFD (*n* = 4)	The level of *A. muciniphila* decreased after a week of administration of an HFD.
Régnier *et al* (2020)^152^	Diet-induced obesity and diabetes	Feces	16S rRNA sequencing, qPCR	1. Control diet (*n* = 9–10)2. High-fat and high-sucrose diet (*n* = 9–10)	The 16S rRNA sequencing result showed the relative abundance of *Akkermansia* had an increased trend in high-fat and high-sucrose group without reaching a statistical significance. However, qPCR analysis showed the copy number of *A. muciniphila* was comparable between two groups.
Wu *et al* (2020)^153^	HFD induced obesity	Cecal sample	16S rRNA sequencing	1. Low fat diet (LFD) (*n* = 12)2. HFD (*n* = 12)	The abundance of *A. muciniphila* was lower in HFD fed mice than that in LFD fed mice.
Rats					
Fåk *et al* (2015)^154^	HFD induced obesity	Cecal sample	qPCR	1. Low fat (*n* = 7) 2. High fat (*n* = 7)	The abundance of *A. muciniphila* was increased with high-fat feeding.
Wang *et al* (2015)^52^	HFD induced obesity	Feces	qPCR	1. Normal (*n* = 8)2. HFD (*n* = 8)	The level of *A. muciniphila* in the HFD-fed rats was lower than that in the normal group rats.
Human					
Santacruz *et al* (2010)^61^	Overweight pregnant women	Feces, at 24 weeks of pregnancy	qPCR	1. Normal-weight (*n* = 34)2. Overweight (*n* = 16)	*A. muciniphila* numbers were lower in women with excessive weight gain than in women with normal weight gain during pregnancy.
Karlsson *et al* (2012)^62^	Overweight and obese children	Feces, at a selected time point	qPCR	1. Normal weight (*n* = 20)2. Overweight (*n* = 20)3. Obesity (*n* = 20)	The abundance of *A. muciniphila* was less in overweight and obese children than that in normal weight children.
Zhang *et al* (2013)^77^	T2DM	Feces	16S rRNA sequencing	1. Normal glucose tolerance (*n* = 44) 2. Pre-diabetes (*n* = 64) 3. Type 2 diabetes (*n* = 13)	The abundance of *A. muciniphila* was reduced in the subjects with pre-diabetes and type 2 diabetes compared to subjects with normal glucose tolerance.
Teixeira *et al* (2013)^59^	Obese females	Feces, at a selected time point	qPCR	1. Lean females (*n* = 17)2. Obese females (*n* = 15)	The level of *A. muciniphlia* was higher in lean females than that in obese females.
Chatelier *et al* (2013)^155^	Obesity	Feces	Metagenomic sequencing	1. Low gene count (LGC) (*n* = 68)2. High gene count (HGC) (*n* = 224)	Obese LGC individuals gained on average significantly more weight than HGC individuals during the past 9 years. The level of *A. muciniphila* decreased in LGC group compared to HGC group.
Escobar *et al* (2014)^56^	Obesity	Feces	16S rRNA sequencing	30 volunteers from Colombians	The level of *Akkermansia* was decreased with the increasing BMI in the Colombian dataset.
Remely *et al* (2015)^156^	Obesity	Feces	qPCR	Obese individuals (*n* = 33)	The level of *A. muciniphila* in obese individuals was higher than that before intervention, after 16-week weight loss diet.
Brahe *et al* (2015)^157^	Postmenopausal women with obesity	Feces	Metagenomic sequencing	53 women with obesity	The species *A. muciniphila* was negatively associated with markers for insulin resistance or dyslipidaemia.
Yassour *et al* (2016)^58^	Obesity and T2DM	Feces, at up to two time points each (12–44 months apart)	Metagenomic sequencing	20 monozygotic Korean twins	The abundance of *A. muciniphila* was negatively correlated with BMI, fasting blood sugar, and insulin levels.
Dao *et al* (2016)^78^	Overweight and Obesity	Feces, at baseline, 6 weeks after calorie restriction and 12 weeks after stable body weight	qPCR	1. Overweight (*n* = 11)2. Obesity (*n = *38)	The abundance of *A. muciniphila* at baseline was negatively correlated with fasting blood glucose, waist-to-hip ratio, and subcutaneous fat cell diameter.
Wang *et al* (2017)^158^	T2DM	Feces	16S rRNA sequencing	1. Patients with short durations of diabetes (*n* = 18)2. Patients with medium durations of diabetes (*n* = 35)3. Patients with long durations of diabetes (*n* = 21)	There was a significantly higher abundance of *A. muciniphila* in patients with short and medium durations than those with long duration of diabetes.
Liu *et al* (2017)^63^	Obesity	Feces	Metagenomic sequencing	1. Lean controls (*n* = 105) 2. Obese individuals (*n* = 95)	*A. muciniphila* were highly enriched in lean controls.
Li *et al* (2017)^159^	Hypertension	Feces	Metagenomic sequencing	1. Healthy controls (*n* = 41)2. pre-hypertension (pHTNs) (*n* = 56)3. Hypertension (HTN) (*n* = 99)	The abundance of *A. muciniphila* was higher in the healthy controls than in the patients with pHTNs and HTN.
Borgo *et al* (2017)^64^	Obese children	Feces	16S rRNA amplification followed by denaturing gradient gel electrophoresis (DGGE) analysis and sequencing, qPCR	1. Obese children (*n* = 28) 2. Normal-weight children (*n* = 33)	The abundance of *A. muciniphila* in obese children was significantly lower than that in normal weight children.
Cuesta-Zuluaga *et al* (2018)^69^	Obese and cardiometabolically abnormal subjects	Feces	16S rRNA sequencing	1. Normal weight subjects (*n* = 138)2. Overweight subjects (*n* = 171)3. Obese subjects (*n* = 132)(According to the BMI categories, each group was divided into cardiometabolically healthy and cardiometabolically abnormal)	*A. muciniphila* has higher prevalence in cardiometabolically healthy and normal weight participants when compared with obese and cardiometabolically abnormal subjects.
Chelakkot *et al* (2018)^41^	T2DM	Feces	Metagenomic sequencing	1. T2DM patients (*n* = 12)2. Healthy controls (*n* = 8)	There are more AmEVs in the fecal samples of healthy controls compared with those of patients with T2DM.
Thingholm *et al* (2019)^65^	Obese individuals with and without T2DM	Feces	16S rRNA sequencing, Metagenomic sequencing	1. Lean non-diabetic (*n* = 95) 2. Obese non-diabetic (*n* = 55) 3. Obese individuals with T2DM (*n* = 51)	The abundance of *A. muciniphila* decreased in obese individuals.
Singh *et al* (2019)^160^	Aging	Feces	16S rRNA sequencing	1. Healthy aging (HA) (*n* = 33)2. Non-healthy aging (NHA) (*n* = 32)	The abundance of *Akkermansia* was lower in the HA group than that in the NHA group.
Salah *et al* (2019)^66^	Obesity and T2DM	Feces	16S rRNA sequencing	1. Controls without obesity or T2DM (*n* = 5)2. Obese adults without T2DM (*n* = 25)3. T2DM without obesity (*n* = 5)4. Adults with both obese and T2DM (*n* = 25)	The abundance of *A. muciniphila* was decreased in obese adults when compared with control individuals.
Mitsou *et al* (2019)^67^	Obesity	Feces	qPCR	1. Normal weight subjects (*n* = 30)2. Overweight/obese subjects (*n* = 80)	Overweight/obese subjects were more prone in low bimodal levels of *A. muciniphila* compared to normal-weight individuals. Low bimodal levels of *A. muciniphila* were positively associated with fasting blood glucose.
Medina-Vera *et al* (2019)^161^	T2DM	Feces	16S rRNA sequencing	1. T2DM patients (*n* = 151)2. Healthy controls (*n* = 50)	The level of *A. muciniphila* decreased in T2DM, compared to healthy subjects.
Dao *et al* (2019)^60^	Obesity	Feces, at 1, 3, and 12 months	Metagenomic sequencing, qPCR	65 women with severe obesity	The relative abundance of *A. muciniphila* was significantly lower in severe obesity than in moderate obesity.
Nistal *et al* (2019)^68^	Patients with nonalcoholic fatty liver disease (NAFLD) associated with obesity	Feces	16S rRNA sequencing	1. Healthy adults (*n* = 20)2. Obese patients with NAFLD (*n* = 36)3. Obese patients without NAFLD (*n* = 17)	When compared to healthy controls, the relative abundance of *A. muciniphila* was reduced in obese patients, both with or without NAFLD.
Marvasti *et al* (2020)^57^	Obesity	Feces	qPCR	1. Normal weight subjects (*n* = 50)2. Obese subjects (*n* = 50)	The abundance of *A. muciniphila* significantly decreased in obese group, compared to the normal weight group.
Liang *et al* (2020)^70^	Obese children	Feces	16S rRNA sequencing	1. Obesity (*n* = 42)2. Normal weight (*n* = 57)	The relative abundance of *A. muciniphila* in children with obesity was higher than that in children with normal-weight.
Journey *et al* (2020)^162^	Obesity	Feces, at the beginning and end of the 2015–2016 academic year	qPCR	42 college freshmen living in dormitories at Arizona State University (24 female and 15 male adolescents)	The abundance of *A. muciniphila* was negatively associated with both the increasement of percent waist circumference and percent body mass index.
Hsu *et al* (2020)^163^	Chronic kidney disease (CKD) (children and adolescents)	Feces	16S rRNA sequencing	1.G1 (*n* = 79)2.G2 (*n* = 27)3.G3 (*n* = 7)4.G4 (*n* = 2)(Participants were categorized according to eGFR (mL/min/1.73 m2): G1 ≥ 90, G2 60–89, G3 30–59, or G4 15–29.)	CKD children with an abnormal ambulatory BP monitoring profile had decreased abundance of *A. muciniphila*.

Given this negative correlation, the role of *A. muciniphila* in obesity has been widely investigated in both mice and human subjects. Everard *et al*. reported that administration of *A. muciniphila* reversed a series of disorders in HFD-fed mice, including reducing body weight, relieving insulin resistance, and fasting hyperglycemia, as well as increasing the mRNA expression of genes involved in the regulation of adipocyte differentiation and lipid oxidation.^19^ From that on, many research teams further found live or even pasteurized *A. muciniphila* as well as its components, including the outer membrane protein Amuc_1100, *A. muciniphila*-derived extracellular vesicles (AmEVs), and secreted protein P9, were effective in improving diet-induced obesity.^19,21,22,24,26,27,29,32–34,36,39,42^ These findings were summarized in Table 2. The benefits of *A. muciniphila* in obesity intervention has also been investigated in one clinical trial. Depommier *et al*. compared the safety and efficacy of live and pasteurized *A. muciniphila* in adults with overweight or obesity. Their results showed that daily oral supplementation of 10^10^ CFU of live or pasteurized *A. muciniphila* for three months was safe and well tolerated. However, improved insulin sensitivity, reduced insulinemia, and plasma total cholesterol was only present in patients given pasteurized *A. muciniphila* supplementation.^37^ This prospective study showed the feasibility to administer *A. muciniphila* to obese humans, however, further study is needed to demonstrate the relationship between supplement of *A. muciniphila* and improvement of metabolic parameters on a larger scale of subjects.

**Table 2.** The efficacy of *A. muciniphila* supplementation on metabolic diseases

Ref.	Bacterial status	Diet	Disease model	Dosage and period		study group	Treatment outcome
Animals							
Everard *et al* (2013)^19^	Live *A. muciniphila*, heat-killed *A. muciniphila*	CT or HFD	HFD-fed obese mice	1. CT and HFD group mice orally administrated with 0.2 ml sterile anaerobic PBS containing a similar end concentration of glycerol (2.5% vol/vol) for 4 weeks.2. CT + AKK and HFD + AKK group mice orally administrated with 2 × 10^8^ CFU/0.2 ml *A. muciniphila* suspended in sterile anaerobic PBS.3. HFD + K-AKK group mice orally administrated with 2 × 10^8^ CFU/0.2 ml heat-killed *A. muciniphila* for 4 weeks.		1. Control diet (CT) (*n* = 10)2. HFD (*n* = 10)3. CT + AKK (*n* = 10)4. HFD + AKK (*n* = 10)5. HFD + K-AKK (*n* = 10)	*A. muciniphila* treatment reversed HFD including fat-mass gain, metabolic endotoxemia, adipose tissue inflammation, and insulin resistance. It also increased the intestinal levels of endocannabinoids and gut peptide secretion. And all these effects required viable *A. muciniphila* because treatment with heat-killed cells did not improve the metabolic profile or the mucus layer thickness.
Shin *et al* (2014)^22^	Live *A. muciniphila*	NCD or HFD	HFD induced obese mice	1. HFD group treated with PBS for the 6 weeks.2. HFD + AKK group treated with 4.0 × 10^8^ CFU *A. muciniphila* for 6 weeks.		1. HFD (*n* = 6)2. HFD + AKK (*n* = 6)	Oral administration of *A. muciniphila* to HFD-fed mice significantly enhanced glucose tolerance and attenuated adipose tissue inflammation by inducing Foxp3 regulatory T cells in the visceral adipose tissue.
Org *et al* (2015)^71^	HFD/high-sucrose diet (HSD) induced obesity	High-fat, high-sucrose (HF/HS) diet	(HF/HS) diet induced obese mice	1. HF/HS-AKK group was treated five times per week with *A. muciniphila* by oral gavage at a dose of 1.44 × 10^9^ CFU/0.2 mL for five weeks. 2. HF/HS group was treated with an oral gavage of an equivalent volume of heat-killed *A. muciniphila* for five weeks.	1. HF/HS-AKK (*n* = 5)2. HF/HS (*n* = 5)		Oral administration of *A. muciniphila* significantly reduced body weight and total body fat as well as improved metabolic parameters in HF/HS fed mice.
Plovier *et al* (2017)^21^	Live *A. muciniphila*, pasteurized *A. muciniphila*, and Amuc_1100	ND or HFD	HFD-induced obese mice	1. Control groups (ND and HFD) were treated with an oral gavage of an equivalent volume of sterile PBS containing 2.5% glycerol for 5 weeks. 2. HFD + AKK M group mice orally administered 2 × 10^8^ CFU/150 μl *A. muciniphila* grown on the mucus-based medium for 5 weeks.3. HFD + AKK S group mice orally administrated 2 × 10^8^ CFU/150 μl *A. muciniphila* grown on the synthetic medium for 5 weeks.4. HFD + AKK P group mice orally administrated 2 × 10^8^ CFU/150 μl pasteurized *A. muciniphila* for 5 weeks.5. Amuc_1100 group mice orally administrated 3 μg of the protein Amuc_1100 for 5 weeks.	1. ND (*n* = 10) 2. HFD (*n* = 9) 3. HFD + AKK M (mucus) (*n* = 9) 4. HFD + AKK S (synthetic) (*n* = 8) 5. HFD + AKK P (pasteurized) (*n* = 9) 6. Amuc_1100 (*n* = 9)		*A. muciniphila* retains its beneficial effects when grown on the synthetic medium. Pasteurization of *A. muciniphila* enhanced its capacity to reduce fat mass development, insulin resistance and dyslipidemia in mice. Amuc_1100 can interact with Toll-like receptor 2 and improve the gut barrier.
Li *et al* (2016)^20^	Live *A. muciniphila*, heat-killed *A. muciniphila*	NCD or WD	Western diet-induced atherosclerosis in *Apoe^−/−^* mice on a C57BL background	1. WD + PBS group treated PBS for 8 weeks.2. WD + AKK group treated 5 × 10^9^ CFU live *A. muciniphila* for 8 weeks. 3. WD + hk-AKK group treated 5 × 10^9^ heat-killed *A. muciniphila* for 8 weeks.	1. NCD (*n* = 8–10)2. WD (*n* = 8–10)3. WD + AKK (*n* = 8–10)4. WD + hk-AKK (*n* = 8–10)5. WD + PBS (*n* = 8–10)		Oral gavage with *A. muciniphila* protected against WD-induced atherosclerotic lesion formation in *Apoe^−/−^* mice. It also ameliorated both aortic and systemic inflammation, decreased intestinal permeability and reduced the penetration of gut-derived lipopolysaccharide into circulation in WD-fed *Apoe^−/−^* mice.
Shen *et al* (2016)^23^	Live *A. muciniphila*	CD	CREBH-null mice	1. WT + Veh and KO + Veh mice treated with PBS that included 25% (vol/vol) glycerol for 2 weeks. 2. WT + AKK and KO + AKK mice treated with AKK for 2 weeks. 3. KO + inactive-AKK mice treated with heat-inactivated AKK for 2 weeks.	1. WT + AKK (*n* = 6–12)2. WT + Veh (*n* = 6–12)3. KO (CREBH-null mice) + AKK (*n* = 6–12)4. KO + Veh (*n* = 6–12)5. KO + inactive-AKK (*n* = 6–12)		*A. muciniphila* administration protected mice from an acute fat load–induced hyperlipidemia compared with vehicle-treated mice. It also significantly ameliorated chronic hypertriglyceridemia, improved insulin sensitivity, and prevented overproduction of postprandial chylomicrons in CREBH-null mice. Treatment with *A. muciniphila* further improved hepatic endoplasmic reticulum stress and metabolic inflammation in CREBH-null mice.
Zhao *et al* (2017)^24^	Live *A. muciniphila*	NCD	NCD induced obese mice	1. NCD + PBS group orally administrated sterile PBS (NCD + PBS) for five weeks. 2. NCD + AKK group orally administrated 2.0 × 10^8^ CFU/200 µl *A. muciniphila* every day for five weeks.	1. NCD + PBS (*n* = 10)2. NCD + AKK (*n* = 10)		*A. muciniphila* supplementation significantly alleviated body weight gain, reduced fat mass and improved glucose tolerance and insulin sensitivity when compared with the vehicle group. It also reduced gene expression related to fatty acid synthesis and transport in liver and muscle as well as alleviated the endoplasmic reticulum stress in liver and muscle. *A. muciniphila* decreased plasma levels of lipopolysaccharide (LPS)-binding protein (LBP) and leptin, inactivated LPS/LBP downstream signaling in liver and muscle as well as increased anti-inflammatory factors.
Gao *et al* (2017)^25^	Live *A. muciniphila*	NCD or HFD	HFD induced metabolic syndrome in mice	1. The NCD + AKK and HFD + AKK groups mice were treated daily with oral doses of 1 × 10^9^ CFU/300 μl *A. muciniphila* for 14 weeks.2. NCD- and HFD-fed control mice received a gavage with the corresponding sterile culture medium for *A. muciniphila* for 14 weeks.	1. NCD (*n* = 6–8)2. HFD (*n* = 6–8)3. NCD + AKK (*n* = 6–8)4. HFD + AKK (*n* = 6–8)		*A. muciniphila* administration altered body composition and energy efficiency, promoted the browning of white fat tissue, and improved the lipid and glucose metabolism disorder in the HFD-fed mice.
Sheng *et al* (2018)^26^	Live *A. muciniphila*	WD	WD induced obese mice	1. WD-fed WT mice + PBS group orally administrated PBS for 1 month.2. WD-fed WT mice + AKK group orally administrated *A. muciniphila* 10^9^ CFU/mouse per day for 1 month.	1. WD-fed WT mice + PBS (*n* = 3–4)2. WD-fed WT mice + AKK (*n* = 3–4)		Supplementation of *A. muciniphila* reduces body weight and regulates lipid metabolism.
Lee *et al* (2018)^27^	AmEVs	HFD	Six-week-old mice were fed a HFD for 39 weeks to induce metabolic disorders, including obesity and T2DM.	1. HFD + PBS mice were orally administrated PBS for 5 weeks.2. HFD + AmEVs were orally administrated 20 µg AmEVs daily for 5 weeks.	1. HFD + PBS (*n* = 4) 2. HFD + AmEVs (*n* = 3)		AmEVs significantly decreased the body weight gain, when compared with HFD-fed group. The total cholesterol level in the AmEVs-fed group was lower than that in the HFD-fed group. The epididymal fat pad weight in the AmEVs-fed group was lower than that in the NC group, albeit not significantly so.
Bodogai *et al* (2018)^40^	Live *A. muciniphila*	/	Young (10 to 12 weeks) and aged (18 to 24 months) female C57BL/6 mice (*n* = 400)	1. Young and aged group mice orally administrated vehicle every second day for 20 days.2. Aged mice + AKK group orally administrated 1 × 10^8^ *A. muciniphila* every second day for 20 days.	1. Young (*n* = 7)2. Aged (*n* = 7)3. Aged mice+AKK (*n* = 7)		Supplementation with *A. muciniphila* could restore normal insulin response in aged mice and macaques.
Chelakkot *et al* (2018)^41^	AmEVs	NCD or HFD	HFD induced a diabetic phenotype	1. NCD and HFD groups treated with PBS for 2 weeks.2. NCD+AmEVs and HFD+AmEVs groups EVs were orally administered for 2 weeks.	1. NCD (*n* = 5–7)2. NCD + AmEVs (*n* = 5–7)3. HFD (*n* = 5–7)4. HFD + AmEVs (*n* = 5–7)		AmEVs improved body weight and glucose tolerance in diabetic mice. It also reduced HFD-induced barrier permeability.
Hänninen *et al* (2018)^28^	Live *A. muciniphila*	/	Non-obese diabetic (NOD) mice	1. Vehicle control group treated with PBS three times a week for 7 weeks.2. *A. muciniphila* group treated with 2 × 10^8^ CFU of *A. muciniphila* three times a week for 7 weeks.	1. Vehicle control (*n* = 25)2. *A. muciniphila* (*n* = 24)		Oral gavage of female NOD/Jax mice with *A. muciniphila* delayed diabetes significantly as compared with gavage with vehicle only.
Shin *et al* (2019)^39^	Live *A. muciniphila*	HFD	HFD induced obese mice	1. HFD group orally administrated with 25% glycerol in sterile PBS for 4 weeks.2. HFD + AKK mucin (+) group orally administrated with *A. muciniphila* (1.0 × 10^8^ CFU/day) grown on mucus-based for 4 weeks.3. HFD + AKK mucin (-) group orally administrated with *A. muciniphila* (1.0 × 10^8^ CFU/day) grown on mucus-depleted medium for 4 weeks.	1. HFD group (*n* = 4–5) 2. HFD + AKK mucin (+) group (*n* = 4–5) 3. HFD + AKK mucin (-) group (*n* = 4–5)		Administration of *A. muciniphila* grown under mucin-depleted conditions to high-fat diet-induced diabetic mice reduced obesity and improved intestinal barrier integrity more efficiently than administration of *A. muciniphila* grown under mucin-containing conditions.
Ashrafian *et al* (2019)^29^	Live *A. muciniphila*, AmEVs	ND or HFD	HFD-induced obese mice	1. ND or HFD-fed mice treated with 200 μl PBS for 5 weeks.2. HFD + Live *A. muciniphila* and ND + Live *A. muciniphila* groups treated with 10^9^ CFU/200 μl live *A. muciniphila*.3. HFD + AmEVs and ND + AmEVs groups treated with 10 μg protein/200 μl AmEVs.	1. HFD + PBS (HPBS) (*n* = 5)2. HFD + Live *A. muciniphila* (*n* = 5)3. HFD + AmEVs (*n* = 5)4. ND + PBS (NPBS) (*n* = 5)5. ND + Live *A. muciniphila* (*n* = 5)6. ND + AmEVs (*n* = 5)		*A. muciniphila* and AmEVs reduced food intake and body weight gain. They can also alleviate adipose Inflammation, ameliorate HFD-induced intestinal barrier dysfunction, regulate inflammation and energy homeostasis in the colon of obese mice, and regulate gene expression involved in FA oxidation and energy metabolism of adipose tissues.
Everard *et al* (2019)^30^	Live *A. muciniphila*	ND or HFD	*Napepld^∆IEC^* mice fed with ND or HFD	1. WT ND, WT HFD and *Napepld^∆IEC^* HFD group mice treated daily with 150 μl PBS.2. WT ND AKK and *Napepld^∆IEC^* HFD AKK groups mice treated daily with an oral gavage of either 2.0 × 10^8^ CFU of *Akkermansia muciniphila*.	1. WT ND (*n* = 8–10)2. WT ND AKK (*n* = 8–10)3. WT HFD (*n* = 8–10)4. *Napepld^∆IE^*^C^ HFD (*n* = 8–10)5. *Napepld ^∆IEC^* HFD AKK (*n* = 8–10)		*Napepld^∆IEC^* mice are hyperphagic upon first HFD exposure, and develop exacerbated obesity and steatosis. *A. muciniphila* administration partly counteracts the gene deletion effects.
Van der Lugt *et al* (2019)^31^	Live *A. muciniphila*	An ad libitum purified diet	Aging *Ercc1* ^−/Δ7^ mice	1. The control group simultaneously received oral gavages PBS. 2. *Ercc1^−/Δ7^* + AKK group mice were supplemented with *Akkermansia muciniphila* by oral gavage at a dose of 2 × 10^8^ CFU/200 μL, three times a week, for a total of 10 weeks. Oral gavages were given in the morning.	1. Control group (*n* = 18)2. *Ercc1^−/Δ7^* + AKK (*n* = 18)		Supplementation with *A. muciniphila* prevented the age-related decline in thickness of the colonic mucus layer and attenuated inflammation and immune-related processes at old age.
Wu *et al* (2020)^72^	An *Akkermansia muciniphila* subtype (*A. muciniphila^sub^*)	NCD or HFD	HFD-induced obesity and diabetes	1. The mice in the HFD + PBS and NCD + PBS groups were administered the PBS vehicle.2. The mice in the HFD + AKK^sub^ and NCD + AKK^sub^ groups were orally administered AKK^sub^ daily at a dose of 10^9^ CFU/200 μl.	1. HFD + PBS (*n* = 10)2. HFD + AKK^sub^ (*n* = 10)3. NCD + PBS (*n* = 10)4. NCD + AKK^sub^ (*n* = 10)		*A. muciniphila^sub^* reduced body weight and food consumption, improved blood glucose control, and prevented memory decay but not depression induced by high fat diet. *A. muciniphila^sub^* can also decrease systemic inflammation and improve tryptophan metabolism in mice fed HFD, produce high concentrations of acetic acid, propionic acid and isovaleric acid, and restore gut microbiota altered by HFD.
Depommier *et al* (2020)^32^	Pasteurized *A. muciniphila*	HFD	HFD-induced obesity	1. ND and HFD groups treated with 180 µl of vehicle solution (PBS containing 2.5% glycerol) for 5 weeks.2. HFD pasteurized *A. muciniphila* group mice were treated daily with an oral gavage of either 2 × 10^8^ CFU/180 µl of pasteurized *A. muciniphila*.	1. ND (*n* = 7)2. HFD (*n* = 7)3. HFD pasteurized *A. muciniphila* (*n* = 7)		Daily oral administration of pasteurized *A. muciniphila* alleviated diet-induced obesity and decreased food energy efficiency. This effect was associated with an increase in energy expenditure and spontaneous physical activity.
Huo *et al* (2020)^33^	Live *A. muciniphila*	ND or HFD	HFD induced obesity	1. The mice in the HFD group treated with 0.9% saline solution for 9 weeks.2. The mice in the HFD + AKK group treated with 10^9^ CFU/kg per day for 9 weeks.	1. ND (*n* = 6)2. HFD (*n* = 6)3. HFD + AKK (*n* = 6)		*A. muciniphila* decreased body weight, relative fat weight, and serum LPS. It can also increase lipid catabolism in epididymal adipose tissues.
Deng *et al* (2020)^42^	*A. muciniphila* I (Amuc_GP01), *A. muciniphila* II (Amuc_GP25)	NCD or HFD	HFD induced obese mice	1. HFD + PBS and NCD + PBS groups mice orally gavaged with 200 µL sterile PBS daily for 16 weeks.2. The remaining groups orally gavaged with *A muciniphila* (5 × 10^9^ CFU/mL) in 200 µL sterile PBS for 16 weeks.	1. HFD + *A. muciniphila* I (*n* = 10)2. HFD + *A. muciniphila* II (*n* = 10)3. HFD + PBS group (*n* = 10)4. NCD + *A. muciniphila* I (*n* = 10)5. NCD + *A. muciniphila* II (*n* = 10)6. NCD + PBS group (*n* = 10)		*A. muciniphila* I and II exert different impacts on blood glucose and lipid metabolism. Both *A. muciniphila* I and II could alleviate brown adipose tissue inflammation and whitening induced by HFD, which were regulated much better under *A. muciniphila* I intervention; *A. muciniphila* I could alleviate endotoxemia in HFD mice while II could not.
Kim *et al* (2020)^34^	Live *A. muciniphila*	NCD or HFD (45% fat diet)	HFD induced obesity in mice	1. The NC + PBS and HFD + PBS groups mice are daily treated with 10^8^ to 10^9^ CFU/ml *A. muciniphila* by oral gavage for 10 weeks.2. Mice in the ND + PBS and HFD + PBS groups were fed with the same volume of PBS by oral gavage for 10 weeks.	1. NC + PBS (*n* = 4)2. NC + AKK (*n* = 4)3. HFD + PBS (*n* = 4)4. HFD + AKK (*n* = 4)		*A. muciniphila* treatment prevented fatty liver disease in obese mice.
Katiraei *et al* (2020)^35^	Live *A. muciniphila*	Atherogenic Western‐type diet containing 1% cholesterol and 0.05% cholate	Hyperlipidemic APOE*3‐Leiden (E3L). CETP mice	1. The AKK group mice are daily treated with 2 × 10^8^ CFU *A. muciniphila* by oral gavage for 4 weeks.2. The Control mice orally gavaged daily for 4 weeks with anaerobic PBS.	1. Control (*n* = 8)2. AKK (*n* = 8)		*A. muciniphila* administration decreased body weight as well as plasma TC and TG levels.
Yoon *et al* (2021)^36^	P9	HFD	HFD induced obese mice	1. HFD group mice treated with 200 μl anaerobic PBS.2. HFD + Am group mice treated with 4.0 × 10^8^ CFU/200 μl *A. muciniphila*.3. HFD + P9 group mice treated with 100 μg per mouse for 8 weeks.	1. HFD (*n* = 7–10)2. HFD + *A. muciniphila* (Am) (*n* = 7–10)3. HFD + P9 (*n* = 7)		*A. muciniphila* increases thermogenesis and glucagon-like peptide-1 (GLP-1) secretion in HFD-induced C57BL/6 J mice by induction of uncoupling protein 1 in brown adipose tissue and systemic GLP-1 secretion. Purified P9 alone is sufficient to induce GLP-1 secretion and brown adipose tissue thermogenesis.
Human							
Depommier *et al* (2019)^37^	Live *A. muciniphila*, pasteurized *A. muciniphila*	/	Overweight/obese insulin resistant	1. Placebo group received placebo per day for 3 months. 2. Alive group received 10^10^ CFU alive *A. muciniphila* per day for 3 months.3. Pasteurized group received 10^10^ pasteurized *A. muciniphila* per day for 3 months.	1. Placebo (*n* = 11)2. Pasteurized (*n* = 12)3. Alive (*n* = 9)		The supplemention of *A. muciniphila* was safe and well-tolerated, it can reduce the levels of relevant blood markers of liver dysfunction and inflammation while the overall gut microbiome structure was unaffected.
Perraudeau *et al* (2021)^38^	Live *A. muciniphila*	/	T2DM	1. Placebo group received colloidal silicon dioxide for 12 weeks.2. WBF-010 group received WBF-010 (which contains inulin, *Clostridium beijerinckii, Clostridium butyricum* and *Bifidobacterium infantis*).3. WBF-011 group received WBF-011 (which contains inulin, *Akkermansia muciniphila, Clostridium beijerinckii, Clostridium butyricum, Bifidobacterium infantis* and *Anaerobutyricum hallii*).	1. Placebo (*n* = 26)2. WBF-010 (*n* = 27)3. WBF-011 (*n* = 23)		Compared with placebo, a statistically significant decrease in total glucose AUC_0-180 min_ was observed in WBF-011 group. Incremental glucose AUC_0-180 min_ was also lower in WBF-011 group. The validated measure of long-term glucose control, A1c, was reduced by 0.6 in WBF-011 group when compared with placebo.

### 2.2. A. muciniphila *and T2DM*

T2DM is a common metabolic disease, which is genetic susceptible and obesity-oriented.^73–75^ In addition to well-recognized genetic and environmental risk factors, gut dysbiosis has emerged as a new risk factor for T2DM development,^2,76^ in which the decreased abundance of *A. muciniphila* was frequently observed in either diabetic mice,^19^ or patients with pre-diabetes or T2DM.^77^ Our previous study demonstrated that administration of *A. muciniphila* reduced the fasting blood glucose level in western diet-fed mice, suggesting that *A. muciniphila* contributes to T2DM recovery.^26^ Moreover, a series of studies have shown that *A. muciniphila* supplementation may regulate host lipoprotein metabolism, improve insulin sensitivity, and alleviate hepatic metabolic inflammation in mice.^23,29,42^ A recent clinical trial of a new probiotic formulation WBF-011, which contains *A. muciniphila* and another four bacterial strains as well as inulin, found that WBF-011 improved postprandial blood glucose in T2DM patients.^38^ This is the first randomized controlled trial to show the effect of *A. muciniphila* on improving T2DM in human subjects. In addition, the baseline abundance of *A. muciniphila* also affected the metabolic outcomes of calorie restriction: individuals with higher baseline *A. muciniphila* showed better responses toward calorie restriction than those who had low baseline *A. muciniphila*.^78^

Interestingly, the alteration of *A. muciniphila* was also found to be involved in the anti-T2DM effect of metformin, a widely used first-line medicine for T2DM.^79^ Shin *et al*. reported that metformin significantly increased the abundance of *A. muciniphila* in HFD-fed mice, while oral supplementation of *A. muciniphila* to HFD-fed mice without metformin also improved glucose tolerance and reduced inflammation in adipose tissue.^22^ Cuesta-Zuluaga *et al*. found higher abundance of *A. muciniphila* in T2DM patients with metformin therapy than healthy subjects (Table 3).^80^ These results suggest that the elevated *A. muciniphila* contributes to the anti-T2DM effect of metformin, providing new understanding on the role of *A. muciniphila*. Overall, existing evidence highlights the significance of *A. muciniphila* in T2DM development, as well as its involvement in the anti-T2DM activity of clinical medicines.

**Table 3.** The interventions aiming at improving metabolic disorders in humans accompanied by the increase of *A. muciniphila.*

Ref.	Disease condition	Intervention	Intervention period	Diet	Study group	Sample type	Sample detection	Beneficial changes achieved
Obesity								
Kim *et al* (2014)^164^	Obesity	Ephedra sinica (Ma Huang)	8 weeks	Subjects maintain usual daily diet, limiting caloric intake to 20–25 kcal/kg, according to subject’s weight.	Subjects should be obese (BMI ≥ 25 kg/m^2^) and female between the ages of 40 and 65 (*n* = 7).	Feces	16S rRNA sequencing	Body weights, body mass index, and body fat percentage of subjects were reduced after intake Ephedra sinica. Negative correlation of *Akkermansia* with waist circumference, body weight, and BMI indicates an association of *Akkermansia* genus with weight loss.
Dao *et al* (2016)^78^	Overweight or obesity	Calorie restriction 112(CR)	12 weeks	CR diet enriched with fibers and protein.	1. AKK LO (*n* = 24)2. AKK HI (*n* = 25)	Feces	qPCR	The Akk HI group remained metabolically healthier throughout the CR intervention when compared with AKK LO. While there was a decrease in *A. muciniphila* abundance in the Akk HI group after CR and the total intervention period, it remained consistently and significantly higher than the Akk LO group.
Palleja *et al* (2016)^165^	Obesity	Roux-en-Y gastric bypass (RYGB)	/	/	13 morbidly obese patients who underwent RYGB	Feces, before RYGB (*n* = 13) and 3 months (*n* = 12) and 12 months (*n* = 8) after RYGB	Metagenomic sequencing	In parallel with the weight loss and metabolic improvements, the abundance of *A. muciniphila* increased within the first 3 months after RYGB and remained high 1 year later.
Payahoo *et al* (2019)^166^	Obesity	Oleoylethanolamide (OEA) supplementation	8 weeks	1. The placebo group who received two capsules containing 125 mg of starch daily similar to the intervention group.2. The OEA group who received 125 mg of OEA daily before lunch and dinner meals.	1. Placebo group (*n* = 30)2. OEA group (*n* = 30)	Feces	qPCR	After eight weeks of OEA supplementation, the abundance of *A. muciniphila* bacterium increased significantly for OEA group compared to placebo group.
Pedret *et al* (2019)^167^	Abdominal obesity	1. Placebo group received 300 mg of maltodextrin.2. Ba8145 group received 100 mg of the live strain, 10^10^ CFU/capsule containing maltodextrin 200 mg.3. H-k Ba8145 group received 100 mg of heat-killed CECT 8145 strain at a concentration of 10^10^ CFU before the heat treatment/capsule containing maltodextrin 200 mg.	3 months	Dietary recommendations were provided according to guidelines of the 2013 Adult Treatment Panel (ATP III). Diet and physical activity were similar among groups, but fiber intake was greater in the h-k Ba8145 group versus the placebo group.	1. Placebo group (*n* = 40)2. Ba8145 group (*n* = 42)3. H-k Ba8145 group (*n* = 44)	Feces	Metagenomic sequencing	Ba8145 decreased the body mass index compared with baseline and placebo group. The decrease in visceral fat area after Ba8145 treatments reached significance only after h-k Ba8145. Ba8145 treatments also increased the incidence of *Akkermansia*. Consistent with this fact, the maximum increase in *Akkermansia* spp. was observed after the Ba8145 live-form administration, when the maximum decrease in BMI occurred. Also, *Akkermansia* content was 1.8% lower in participants over 90 kg after Ba8145 treatments.
Zhang *et al* (2019)^168^	Normal body weight	Resistant starch (RS)	4 weeks	HAM-RS2 (Ingredion Inc., Bridgewater, NJ, USA) at 255.4 kcal/day (2.8 kcal/g, 91.2 g, containing 40 g of RS)	1. Resistant starch (*n* = 19 2. Control starch (*n* = 19)	Feces	16S rRNA sequencing	RS was found to reduce abdominal adiposity in normal-weight subjects. Intra-abdominal visceral and abdominal subcutaneous fat were significantly reduced by taking RS at 40 g/d in the 4-week study. Moreover, an increasing trend of abundance of *Akkermania* was observed after RS intake compared to that at baseline.
Dong *et al* (2020)^169^	Obesity	A dietary intervention trial of overweight and obese subjects who were randomized to a calorie-restricted high protein diet (HPD) (30% calorie intake) or calorie-restricted normal protein diet (NPD) (15%) for 8 weeks.	8 weeks	An HPD (30% protein, 40% carbohydrate, 30% fat by calorie intake) or an NPD (15% protein, 55% carbohydrate, 30% fat)	1. HPD (*n* = 31)2. NPD (*n* = 29)	Feces, at baseline, week 1, week 2, week 4, week 6, and week 8	16S rRNA sequencing	At the end of 8 weeks, the HPD lost more weight on average than the NPD group, though the results were not statistically significant. The three genera with the highest relative abundance were *Akkermansia, Bifidobacterium*, and *Prevotella_9. Akkermansia spp*. and *Bifidobacterium spp*. were elevated at 8-weeks as compared to baseline.
T2DM								
Cortez *et al* (2018)^170^	T2DM	Duodenal-jejunal bypass surgery with minimal gastric resection	12 months	The diet was formulated using Total Energy Expenditure data according to the Cunningham Equation.	1. Duodenal-jejunal bypass surgery with minimal gastric resection (DJBm) (*n* = 11)2. Standard care group (*n* = 11)	Feces, before the operation and after 6 and 12 months (DJBm group), at baseline and after 6 and 12 months (Standard care group)	16S rRNA sequencing	The abundance of *Akkermansia* was increased in duodenal-jejunal bypass surgery with minimal gastric resection group compared with that in the standard care group.
Wu *et al* (2017)^79^	T2DM	Metformin	4 months	A calorie-restricted diet	1. Placebo group (*n* = 18)2. Metformin group (*n* = 22)	Feces	Metagenomic sequencing	Metformin treatment promotes the growth of *A. muciniphila* in vitro. The abundance of *A. muciniphila* was increased in individuals who received metformin for 4 months.
Cuesta-Zuluaga *et al* (2017)^80^	T2DM	Metformin	/	Dietary intake was evaluated through 24-h dietary recalls.	1. T2DM using metformin (T2D-met+) (*n* = 14)2. T2DM not using metformin (T2D-met−) (*n* = 14)	Feces	16S rRNA sequencing	Compared with participants without diabetes, participants with diabetes taking metformin had higher relative abundance of *A. muciniphila*.
Wang *et al* (2018)^158^	T2DM	Liraglutide	42 days	Subjects were on the diet recommended by their primary care physician.	1. Metformin (*n* = 18)2. Liraglutide (*n* = 19)	Feces	16S rRNA sequencing	At baseline, the genus *Akkermansia* showed a significant increase in liraglutide relative to metformin subjects. At Day 42, when controlling for placebo treatments in the statistical model, a significant increase in *Akkermansia* were observed in liraglutide relative to metformin subjects.
Guevara-Cruz *et al* (2019)^171^	Metabolic syndrome (MetS)	A lifestyle intervention with functional foods and energy reduction (−500 kcal) for 75 days	75 days	A low‐saturated‐fat diet, reduced‐energy intake, with functional foods	1. Class III obesity (OCIII)+MetS+functional foods (FF) (*n* = 18)2. Class III obesity (OCIII)+MetS+placebo (P) (*n* = 17)	Feces	16S rRNA sequencing	The level of *A. muciniphila* was increased after intervened with FF.
Shin *et al* (2020)^172^	T2DM	*Scutellaria baicalensis* (SB) combined with metformin	20 weeks	/	1. SB (*n* = 6)2. Placebo (*n* = 6)	Feces	16s rRNA sequencing	SB with metformin treatment may improve the glucose tolerance and inflammation and influence the gut microbiota community in T2DM, the level of *Akkermansia* remarkable increased after SB treatment.

### 2.3. A. muciniphila *and CVD*

CVD remains the leading cause of death worldwide, especially in western countries.^81,82^ The relationship between gut microbiota dysbiosis and CVD has been well determined.^83,84^ Dietary phosphatidylcholine or L-carnitine can be metabolized into trimethylamine (TMA) by the gut microbiota,^85–87^ and then transported to the liver, where TMA is converted into trimethylamine N-oxide (TMAO) by hepatic flavin monooxygenase 3 (FMO3).^88–90^ TMAO has been shown to be a potent trigger and biomarker for CVD.^91,92^ Recently, Plovier *et al*. reported that supplementation with *A. muciniphila* significantly increased the excretion of TMAO and TMA in urine, resulting in decreased plasma TMAO and TMA levels.^21^ In addition, they found that HFD induced two-fold higher FMO3 expression compared with that in control-diet fed mice, whereas treatment with pasteurized *A. muciniphila* could offset this change, suggesting pasteurized *A. muciniphila* intervention may also reduce TMAO production.^21^ Li *et al*. discovered that oral administration of live *A. muciniphila* reduced exacerbation of atherosclerotic lesion formation, as well as aortic and systemic inflammation induced by a western diet, and improved intestinal integrity in antherosclerotic *Apoe^−/−^* mice.^20^ These evidences indicate that *A. muciniphila*, live or pasteurized, has a protective effect against CVD development.

In addition to the co-metabolized TMA/TMAO pathway by host and gut microbiota, short-chain fatty acids (SCFAs), which can be generated by *A. muciniphila*, are also essential metabolites for bridging the crosstalk between *A. muciniphila* and host.^93,94^ The beneficial effects of SCFAs on host metabolism have been extensively investigated and reviewed in CVD.^95,96^ In summary, *A. muciniphila* may play a protective role in CVD development directly or through producing metabolites, and via crosstalk with host and commensal bacteria as well.

### 2.4. A. muciniphila *and NAFLD*

NAFLD is a chronic liver disease and hepatic manifestation of metabolic syndrome. The homeostasis of commensal bacteria and bacteria-derived molecules have been increasingly recognized as a key determinant of NAFLD.^6^ The association of *A. muciniphila* with NAFLD development was recently investigated in obese mice with NAFLD, in which a decreased abundance of *A. muciniphila* was observed.^97^ The administration of anti-obesity drug, such as liraglutide, decreased the levels of total cholesterol and triacylglycerol in the liver while increasing the abundance of *A. muciniphila*.^98^ *A. muciniphila* supplementation also decreased the levels of serum alanine aminotransferase (ALT) and aspartate aminotransferase (AST), and alleviated liver histopathological damage in a mouse model .^99^ Kim *et al*. recently reported that oral administration of *A. muciniphila* prevented fatty liver disease by regulating the expression of genes that regulate fat synthesis and inflammation in the liver.^34^ Moreover, different genotypes of *A. muciniphila*, isolated from human stool samples, played different roles in HFD-induced hyperlipidemia and liver steatosis. Specifically, *A. muciniphila* I (Amuc_GP01, strain GP01 of *A. muciniphila* I) was more effective for alleviating hyperlipidemia, liver steatosis, and glucose tolerance than *A. muciniphila* II (Amuc_GP25, strain GP25 of *A. muciniphila* II) in dietary obese mice. Both two genotypes could improve the intestinal barrier, but the effect of *A. muciniphila* II on improving endotoxemia was not apparent, possibly because they have different characteristics of genes and functions, leading to the identification of specific target pathways and disparate roles.^42^ Overall, these results indicate that *A. muciniphila* may alleviate NAFLD by regulating lipid metabolism and reducing inflammation.

## 3. Mechanisms of *A. muciniphila* action on metabolic diseases

### 3.1. Production of SCFAs and cross-feeding with butyrate-producing bacteria

SCFAs, mainly acetate, propionate, and butyrate, are the principal products of carbohydrate and protein fermentation by gut microbiota.^100^ There are a large number of investigations on the diverse roles of SCFAs in host metabolism.^101,102^ *A. muciniphila* is also a potent generator of acetate, propionate, and oligosaccharides by fermenting mucin,^13,18^ resulting in the activation of fatty acid receptors FFAR2/GPR41 and FFAR3/GPR43.^103^ Interestingly, GPR41 is involved in the microbiota-associated adiposity process, as regularly raised *Gpr41*^−/−^ mice are leaner than wild-type mice, whereas this difference is not observed under germ-free (GF) conditions.^104^ Activation of GPR41 and GPR43 induces intestinal L cells to produce peptide YY (PYY), glucagon-like peptide-1 (GLP-1), and glucagon-like peptide-2 (GLP-2).^105–107^ PYY acts on the gastrointestinal tract by modulating a series of physiological actions. It is a satiety signal released following meals, and decreasing food intake.^108^ GLP-1, one of the principal incretin hormones, promotes glucose-dependent insulinotropic activity, inhibits appetite and food intake, delays gastric emptying, and restores the impaired “incretin effect” in T2DM patients.^108–110^ Acetate could also promote anti-lipolytic activity through GPR43 in white adipose tissue (WAT).^111^ GPR43 stimulation by acetate in the WAT, rather than muscle or liver, also improves glucose and lipid metabolism.^112^ Propionate can be converted into glucose by intestinal gluconeogenesis (IGN), resulting in satiety and reduced hepatic glucose production.^113^ In addition, Lukovac *et al*. found that many transcription factors regulating lipid metabolism and proliferation, such as Hnf4α and p53 family members (Tp53 and Tp73), were affected by both *A. muciniphila* and propionate.^114^

The biological function of *A. muciniphila* is also associated with cross-feeding activity with other butyrate-producing bacteria such as *Faecalibacterium prausnitzii* and *Anaerostipes caccae*, resulting in the increased production of butyrate.^115,116^ Moreover, acetate can also stimulate the growth of butyrate-producing bacteria within the same mucosal niche.^117^ Butyrate is not only a preferred energy source for colon cells,^118^ it also has various beneficial functions for the host, especially in metabolic diseases,^119–121^ and is a more potent agonist for GPR41 than acetate or propionate.^103^

Overall, considering the facts that pasteurization of *A. muciniphila* enhanced its capacity to improve body weight, reduce fat mass development and dyslipidemia,^21^ and administration of Amuc_1100, AmEVs, and secreted P9 protein replicated part of the biological functions of live bacteria (Table 2), the beneficial effects of *A. muciniphila* might depend in part on its capacity of SCFAs production, as well as the cross-feeding relationship with other butyrate-producing bacteria in the gut.

### 3.2. Maintaining the integrity of gut barrier

A number of studies have revealed the correlation between obesity-related metabolic diseases and increased gut permeability, which induces metabolic endotoxemia and inflammation.^122–125^ *A. muciniphila* can considerably improve gut barrier integrity in obese mice by restoring the thickness of the intestinal mucus layer,^19^ and oral supplementation of *A. muciniphila* can increase the number of goblet cells, normalize the mucus thickness of the inner layer, and increase the expression of tight-junction proteins in the gut of both HFD-induced obese mice and mice with alcoholic fatty liver.^19,41,126^ Moreover, Li *et al*. discovered that *A. muciniphila* reduced intestinal permeability by increasing the expression of occludin and ZO-1 in *Apoe^−/−^* mice.^20^ Similarly, Zhao *et al*. reported that administration of *A. muciniphila* could reduce chronic low-grade inflammation by decreasing the permeability of the gut and lipopolysaccharide (LPS)-binding protein (LBP) downstream signaling in the liver and muscle.^24^ Reunanen *et al*. found that *A. muciniphila* could adhere to the intestinal epithelium and enhance enterocyte monolayer integrity *in vitro*, suggesting the ability of *A. muciniphila* to repair the damaged gut barrier.^14^ Furthermore, a large number of studies have shown that bacteria-derived SCFAs maintain the integrity of the intestinal tract and prevent the translocation of LPS across the intestinal wall to alleviate the systemic inflammatory response.^63,127,128^ Not only live *A. muciniphila*, but also pasteurized *A. muciniphila* has been found to enhance the gut barrier function, leading to attenuation of metabolic endotoxemia.^21^ In addition, Amuc_1100 elevated the development of transepithelial electrical resistance in Caco2-cells and increased the expression of tight junction genes in HFD-fed mice to improve intestinal barrier function.^21,129^ Moreover, administration of AmEVs also protected mice from HFD-induced leaky gut (Table 2).^29,41^

In addition, gut barrier function is disrupted in inflammatory bowel disease (IBD). As a mucin-degrader, *A. muciniphila* decreased significantly in dextran sulfate sodium (DSS)-induced colitis in mice and in IBD patients,^130,131^ while administration of *A. muciniphila* or AmEVs have been reported to protect the progression of DSS-induced colitis.^132–135^ On the contrary, the abundance of *A. muciniphila* was found to be increased in the spontaneous colitis in the *Il10*^−/−^ mice model of IBD, while supplementation of *A. muciniphila* further promoted colitis in this model.^136^ However, it should be noticed that these findings in immune compromised or genetic editing mouse models cannot be translated into the human situation directly. Although the role of *A. muciniphila* in colitis is somewhat contradictory based on these reports, most studies supported the potential benefits of *A. muciniphila* or its derived metabolites in respect to their functions of reducing metabolic endotoxemia and systemic inflammation of host, or improving the integrity of gut barrier. Further studies are also needed to determine the exact role of *A. muciniphila*, live or pasteurized, or its metabolites, in colitis.

## 4. Active components of *A. muciniphila* on metabolic diseases

### 4.1. *Pasteurized* A. muciniphila *in metabolic disease*

Although the beneficial effects of *A. muciniphila* on metabolic diseases have been extensively investigated,^19,20,78^ their clinical application is still challenging, owing to its microaerophilic requirements and the loss of activity after heat-killing.^19^ Meanwhile, its growth media probably contain animal-derived compounds, which may have viruses, allergens or bacterial contaminants, thus limiting the usage for clinical study. Plovier *et al*. showed that *A. muciniphila* retained its efficacy in improving metabolic disorders when grown on a synthetic medium, a replacement for animal derived mucins.^21^ Ottman *et al*. identified 79 putative outer membrane and membrane-associated extracellular proteins and 23 of those had different abundance between cells of *A. muciniphila* grown on mucin-containing media and those grown on the non-mucus glucose-containing media.^137^ Moreover, Shin *et al*. found that *A. muciniphila* grown under mucin-containing media upregulated genes encoding mucin-degrading enzymes. In contrast, *A. muciniphila* grown under mucin-depleted conditions upregulated the genes involved in glycolysis, energy metabolic pathways, and 79 genes encoding extracellular protein candidates including Amuc_1100, which, in turn, reduced obesity and improved intestinal barrier more efficiently than administration of *A. muciniphila* grown under mucin-containing conditions.^39^ These findings by different teams suggest mucin in the medium might affect the expression of outer membrane protein and subsequently influence the function of *A. muciniphila*. Interestingly, the recent study discovered that pasteurized *A. muciniphila* was more potent than live *A. muciniphila* for reducing body weight and improving glucose tolerance in HFD-induced obese mice.^21^ This is of great significance for clinical applications, and therefore, increased attention has been paid to the effect of pasteurized *A. muciniphila* on metabolic diseases in recent years. Zhang *et al*. found that oral administration of live or pasteurized *A. muciniphila* significantly increased the levels of plasma high-density lipoprotein (HDL) and decrease hepatic glycogen, as well as reduced inflammatory markers of LPS and TNF-α to alleviate systemic inflammation. However, oral administration of live or pasteurized *A. muciniphila* did not improve glucose levels in diabetic rats.^138^ Depommier *et al*. reported that daily oral supplementation of pasteurized *A. muciniphila* in a small number of subjects improved insulin sensitivity and decreased insulinemia and plasma total cholesterol compared to the placebo group, and the effects of pasteurized *A. muciniphila* were better than those of live *A. muciniphila*.^37^ Although this study only included a small number of participants, the results highlight the potential of pasteurized *A. muciniphila* in clinical applications. It is hypothesized that the effects of pasteurized *A. muciniphila* are attributed to increased energy excretion in feces, reduced carbohydrate absorption, and enhanced intestinal epithelial turnover, but without impacts on intestinal lipid absorption or chylomicron synthesis.^32^ In conclusion, these studies demonstrate that pasteurized *A. muciniphila* is superior to live ones for improving metabolic disorders in mice, rats, and humans; however, the underlying mechanism warrants further investigation.

### 4.2. A. muciniphila *outer membrane protein enhances the gut barrier*

In addition to the *A. muciniphila* itself, the identification of active components of *A. muciniphila* for the treatment of metabolic diseases is also valued recently. Cell derived fragments of *A. muciniphila* have been shown to activate Toll-like receptor 2 (TLR2), in which a highly abundant outer membrane pili-like protein of *A. muciniphila*, named Amuc_1100, with specific activating capacity for TLR2 has been identified by proteomics. TLRs regulate bacterial recognition, intestinal homeostasis, and shape host metabolism.^129^ Ottman *et al*. found that Amuc_1100 activated TLR 2 and TLR4 and significantly increased transepithelial electrical resistance *in vitro*.^129^ In line with the enhanced effects of pasteurized *A. muciniphila*, Amuc_1100 was found to be active after pasteurization.^21^ It has also been found that the expression of *Cnr1*, which codes cannabinoid receptor 1 (CB1) in the jejunum, was lower in Amuc_1100 treated mice.^21^ The downregulation of CB1 was associated with improved gut integrity and lipid accumulation induced by LPS in both liver and adipose tissue.^139^ Therefore, Amuc_1100 might contribute in part to the beneficial effect of live or pasteurized *A. muciniphila* on gut barrier function.

In addition to Amuc_1100, several other proteins of *A. muciniphila* have also been identified including Amuc_1434, Amuc_1686, Amuc_0771, and Amuc_1666. Meng *et al*. reported that Amuc_1434, a member of the aspartic protease family,^140^ degraded mucin2 protein secreted by LS174T and suppresses LS174T cell viability,^141^ suggesting its potential involvement in controlling colon cancer. Nevertheless, the roles of some β-galactosidases with mucin degradation capacity, such as Amuc_1686, Amuc_0771, and Amuc_1666, in regulating metabolic disorders remain unclear so far.^142,143^

### 4.3. A. muciniphila*-derived extracellular vesicles (AmEVs) improve metabolic disorders*

Emerging evidence shows that bacteria-derived extracellular vesicles, especially AmEVs, play important roles in mediating host-bacteria interactions.^41,144,145^ Chelakkot *et al*. analyzed the fecal extracellular vesicles of healthy people and individuals with obesity, and discovered that the feces of healthy individuals contained higher levels of AmEVs than individuals with obesity. They also revealed that oral gavage of AmEVs decreased HFD-induced body weight gain and fat mass, and improved metabolic functions and gut integrity.^41^ Ashrafian *et al*. also found that AmEVs ameliorated intestinal barrier impairment in obese mice.^29^ Moreover, AmEVs regulated inflammation and energy homeostasis in the colon of obese mice. Compared with *A. muciniphila*, oral gavage of AmEVs (10 μg/mouse) alleviated more body and fat weight gain as well as blood glucose and cholesterol levels in HFD-induced obese mice. In addition, AmEVs administration significantly reduced the expression of TLR-4 and induced lower TLR-2 expression in the colon tissue of obese mice.^29^ It is, however, improtant to note that AmEVs administration reducd daily food intake in this study. Additionally, the dose of AmEVs derived from how many *A. muciniphila* is unclear, and the relevant of the oral dose of AmEVs to physiological levels of AmEVs secreted by *A. muciniphila* in the gut is also unknown. Thus, the rationale for the dose used for AmEVs administration need to be further explored. Moreover, whether AmEVs contain Amuc_1100 or other effectors need further investigation. It has been reported that oral administration of AmEVs alleviated DSS-induced inflammatory bowel disease, characterized by reduced infiltration of inflammatory cells through the colon wall.^132^ Overall, these results suggest that AmEVs may protect the host by decreasing intestinal permeability and reducing inflammation in the gut. Therefore, the beneficial effects of the live bacteria may be, at least partly, due to AmEVs.

### 4.4. A. muciniphila*-secreted protein ameliorates metabolic disease*

Since administration of either the cell-free supernatantlive or live *A. muciniphila*, but not bacterial pellet, increased systemic GLP-1 secretion, Yoon *et al*. identified an 84 kDa protein in the culture supernatant, named P9, which accounts for the induction of GLP-1 secretion in HFD-fed mice and L cells.^36^ Administration of P9 to HFD-fed mice prevented obesity and improved glucose tolerance by regulating GLP-1 secretion and inducing brown adipose tissue thermogenesis. In terms of mechanism, ICAM-2 can bind to P9 and modulate P9-induced secretion of GLP-1. In addition, P9 strongly induced IL-6 expression and IL-6 dose-dependently increased GLP-1 secretion, wheares IL-6 deficiency downregulated the expression of ICAM-2 and blocked the response toward P9-induced GLP-1 secretion in mice, demostrating that P9 may improve metabolic diseases through an IL-6-GLP-1 signaling axis.^36^ Since pasteurized *A. muciniphila* and Amuc_1100 also have beneficial effects on regulating blood glucose,^21,32^ P9 is not the only way for this bacterium to regulate glucose homeostasis. Cani and Knauf commented on this research and raised several important questions, such as how P9 acts on L cells to stimulate GLP-1 secretion and whether P9 is specific to *A. muciniphila*, etc.^146^ Further, in addtion to stimulating GLP-1 secretion, whether P9-mediated induction of IL-6 promotes inflammtaion in the gut and how it affects gut barrier function need to be illustrated.

## 5. Conclusions and perspectives

**Figure 1.**
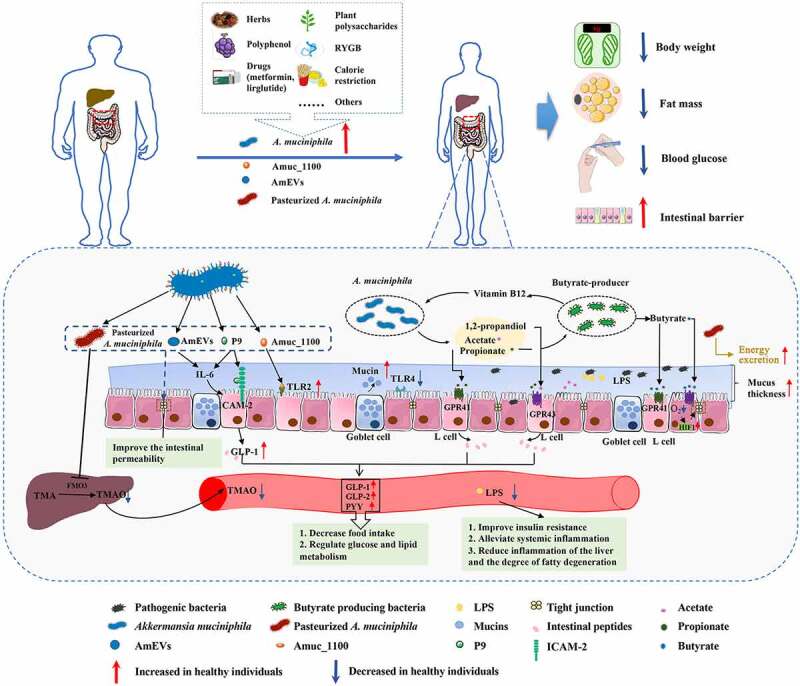
Effects of *Akkermansia muciniphila* and its derived parts on ameliorating metabolic disorders. The level of *A. muciniphila* decreased in several metabolic diseases, including obesity, type 2 diabetes mellitus (T2DM), cardiovascular diseases (CVD), and nonalcoholic fatty liver disease (NAFLD). Many interventions based on the diet and surgery have been reported for improving the human health in context of metabolic disorder, which accompanied by the increase of *A. muciniphila. A. muciniphila* and its different parts, including live or pasteurized *A. muciniphila*, Amuc_1100, P9, as well as AmEVs, have shown to reduce body weight and fat mass gain, and regulate glucose homeostasis and intestinal barrier. Mechanistically, *A. muciniphila* and its different parts have shown to improve the intestinal barrier through up-regulating the expression of tight-junction proteins and reducing the leakage of LPS, thus reducing inflammation. In addition, live *A. muciniphila* produces acetate, propionate, and 1,2-propandiol through the fermentation of mucin. It has a nutritional interaction with butyrate-producing bacteria to stimulate the production of butyrate. These SCFAs can active GPR41 and GPR43 to affect glucose and lipid metabolism. The activation of GPR41 and GPR43 induces the intestinal L cells producing peptide YY (PYY), glucagon-like peptide-1 (GLP-1), and glucagon-like peptide-2 (GLP-2) to decrease food intake. Butyrate promotes the epithelial barrier function by increasing the expression of hypoxia-inducible factor-1α (HIF-1α). Moreover, *A. muciniphila*, live or pasteurized, can normalize the mucus thickness and increase the number of goblet cells. Pasteurized *A. muciniphila* specifically decreases the expression of hepatic flavin monooxygenase 3 (FMO3), increases the excretion of TMAO and TMA in urine, and decreases the level of plasma TMAO. It may also increase fecal energy excretion to reduce obesity. Amuc_1100 can act on TLR2 to regulate intestinal homeostasis. Furthermore, the newly identified secreted protein P9 can bind to ICAM-2 to trigger the secretion of GLP-1 by L cells. Both P9 and AmEVs can simulate IL-6, leading to further secretion of GLP-1. The above mechanism was summarized based on existing articles; however, there may be other mechanisms

The current focus on improving health using gut microbiota-targeted strategies is overwhelming in the context of accumulating experimental and clinical evidence. *A. muciniphila* has emerged as a uniquely promising “next-generation beneficial microbe”, especially for metabolic disease management. A large number of studies have confirmed the alteration of *A. muciniphila* in both animal models and human patients with metabolic diseases (summarized in Table 1), its therapeutic benefits (summarized in Table 2), as well as the efficiency of interventions to boost its abundance (summarized in Table 3). However, most current animal studies with *A. muciniphila* supplementation were performed with *A. muciniphila* grown under mucin-containing conditions. The animal-derived mucin may introduce contaminants and cause compromised beneficial effect of *A. muciniphila* on alleviating metabolic diseases. Therefore, mucin-depleted media should be explored and given more attention for both animal and human investigations. Notably, most of the mechanistic studies on the effects of *A. muciniphila* were performed in animal models. Given the differences between animal models and humans in genetic and environmental elements, it is critical to investigate the real effects and mechanisms of *A. muciniphila* in clinical study. Recently, two randomized controlled trials confirmed that administration of *A. muciniphila* or *A. muciniphila* containing formulation WBF-011 to human subjects with obesity or T2DM were safe and well tolerated in a 12-week period with significant improvement in several metabolic paramaters.^37,38^ This paves the way for more clinical applications of *A. muciniphila* in the near future. The mechanisms underlying the effects of *A. muciniphila* on metabolic diseases have been extensively investigated and are summarized in Figure 1.

Given the similar, or even superior efficacy of pasteurized *A. muciniphila* and its outer membrane proteins such as Amuc_1100 or extracellular vesicles and secreted proteins, the exact mechanisms of *A. muciniphila* activity in the real world of complicated commensal systems is only beginning to be discovered. In this sense, we envisage several critical aspects for future studies on *A. muciniphila*. First, a complete understanding is required with regards to the common or differential mechanisms between live *A. muciniphila* and its derived products, including pasteurized *A. muciniphila*, or the active components such as proteins, vesicles, or metabolites released from the bacteria. Since it is unclear the equivalence of the doses used for AmEVs, P9, and other effectors with the physiological levels of *A. muciniphila* found in the gut, the physiological relevance of exact mechanisms of *A. muciniphila* activity need further exploration. Second, more efforts should be focused on elucidating the complex crosstalk between *A. muciniphila* and commensal bacteria, which may help to explain the discrepant results that have been observed in preclinical and clinical studies. Third, a deeper exploration of the relationship between the specificity in various conditions and the strains of *A. muciniphila*, rather than at the species level. Finally, scientists should always hold a reasonable dose of expectation and skepticism in terms of the overwhelming “good effects” of any potential beneficial microbe, including *A. muciniphila*, if the scientific basis has not been well established. Altogether, given the accumulating evidence of *A. muciniphila* on ameliorating metabolic disorders in both animals and humans, *A. muciniphila* is widely supposed to be one of the most promising microbes with multiple benefits for host metabolism. Although the mechanisms underlying the effects of *A. muciniphila* are largely unclear, identification and isolation of specific effectors and biomolecules that derived from *A. muciniphila* will pave the way for understanding the mechanisms of their action, which is essential and full of challenges for translation of the positive findings in animals to clinic application.
